# Volatile Organic Compounds Frequently Identified after Hyperbaric Hyperoxic Exposure: The VAPOR Library

**DOI:** 10.3390/metabo12050470

**Published:** 2022-05-23

**Authors:** Feiko J. M. de Jong, Paul Brinkman, Thijs T. Wingelaar, Pieter-Jan A. M. van Ooij, Rob A. van Hulst

**Affiliations:** 1 Royal Netherlands Navy Diving and Submarine Medical Centre, 1780 CA Den Helder, The Netherlands; tt.wingelaar@mindef.nl (T.T.W.); pjam.v.ooij.01@mindef.nl (P.-J.A.M.v.O.); 2 Department of Anesthesiology, Amsterdam UMC, Location AMC, 1100 DD Amsterdam, The Netherlands; r.a.vanhulst@amsterdamumc.nl; 3 Department of Respiratory Medicine, Amsterdam UMC, Location AMC, University of Amsterdam, 1100 DD Amsterdam, The Netherlands; p.brinkman@amsterdamumc.nl

**Keywords:** hyperbaric oxygen therapy, hyperoxia, diving and hyperbaric medicine, pulmonary oxygen toxicity, VOC, exhaled breath markers, GC-MS

## Abstract

Diving or hyperbaric oxygen therapy with increased partial pressures of oxygen (pO_2_) can have adverse effects such as central nervous system oxygen toxicity or pulmonary oxygen toxicity (POT). Prevention of POT has been a topic of interest for several decades. One of the most promising techniques to determine early signs of POT is the analysis of volatile organic compounds (VOCs) in exhaled breath. We reanalyzed the data of five studies to compose a library of potential exhaled markers for the early detection of POT. GC-MS data from five hyperbaric hyperoxic studies were collected. Wilcoxon signed-rank tests were used to compare baseline- and postexposure measurements; all ion fragments that significantly varied were compared by similarity using the National Institute of Standards and Technology (NIST) library. All identified molecules were cross-referenced with open-source databases and other scientific publications on VOCs to exclude compounds that occurred as a result of contamination, and to identify the compounds most likely to occur due to hyperbaric hyperoxic exposure. After identification and removal of contaminants, 29 compounds were included in the library. This library of hyperbaric hyperoxic-related VOCs can help to advance the development of an early noninvasive marker of POT. It enables validation by others who use more targeted MS-related techniques, instead of full-scale GC-MS, for their exhaled VOC research.

## 1. Introduction

Breathing gas mixtures with increased partial pressure of oxygen (pO_2_) can result in various side effects. In particular, diving or hyperbaric oxygen therapy can induce adverse effects such as central nervous system oxygen toxicity or pulmonary oxygen toxicity (POT) [1]. This toxicity is likely caused by the damaging effect of reactive oxygen species and other free radicals in the tissues of the lower respiratory tract and can lead to tracheitis and alveolitis [2,3,4,5]. Although POT is initially fully reversible, it can result in pulmonary fibrosis if exposure to an increased pO_2_ continues, or if insufficient recuperation time is given to the affected individuals [6]. Preventing POT in diving professionals such as military or commercial divers, as well as patients receiving hyperbaric oxygen therapy, has been an active area of research for several decades. However, early detection of POT is difficult as pulmonary function testing, the current gold standard, is not accurate enough to detect subtle changes associated with (sub)clinical POT [7].

Several studies have been published recently that use gas chromatography-mass spectrometry (GC-MS) analysis of exhaled breath samples after various hyperbaric hyperoxic exposures with different pO_2_ to detect POT in healthy volunteers [8,9,10,11,12,13]. Although this strategy is feasible, this exploratory and full-scale approach can be cumbersome and results between different studies are often difficult to compare due to differences in wet-lab and dry-lab analysis.

Recently, two extensive meta-analyses with impressively large libraries of VOCs from humans have been published [14,15]. Although there is a large number of collected compounds, they are not subdivided into different categories such as associations with certain organ systems, disease classes, or types of exposures; therefore, their practicability for detecting hyperoxic damage is limited. Thus, composing a library of VOCs frequently found after hyperbaric hyperoxic exposure would greatly facilitate the future identification of breath samples, and pave the way for standardized or even automated VOC analysis for signs of POT. This library can greatly reduce the resources required for analysis and identification of VOC in breath samples, since targeted studies on these metabolites are less burdensome than full-scale analysis of the entire volatilome.

## 2. Results

After Wilcoxon signed-rank testing and analysis of the chromatogram peaks, 41 unique VOCs were identified within the data of the five studies. A total of 12 compounds were considered contaminants, of unclear biological origin, or nonhuman after further analysis. These excluded compounds are listed in Table 1. 

A total of 29 VOCs were retained and listed in the VAPOR library in Table 2. Although not explicitly mentioned in the library, all listed compounds are associated in the literature with pulmonary pathology, cell membrane destruction, immune responses, or various inflammatory diseases.

As mentioned in the Materials and methods section, some ion peaks could not be identified with sufficient certainty. These unidentified VOCs are nominated in Table 3 with their probable carbon length. 

The most frequently found human-associated compounds in the included studies are straight-chain alkanes (n=11), followed by methyl alkanes (n = 6), esters (n = 3), alkenes (n = 2), cycloalkanes (n = 2), aldehydes (n = 2), and other compounds (n = 3). These molecule classes were found in all included studies and seem to be unrelated to the type of hyperbaric exposure or composition of breathing gases used.

There were substantial similarities in VOCs identified in the five included studies. Only four compounds (propyl acetate, 2-butyl-1-octanol, 3-[(1,1-dimethylethoxy)methyl]heptane, and 3,7-dimethyldecane) were found in just one study; the other twenty-five compounds were identified in multiple studies. Eight compounds (nonane, 3-methylnonane, decane, 3-methylheptane, butyl acetate, methylcyclohexane, nonanal, and tridecane) were found in four out of five studies. There were no compounds that were found in every study separately; this can indicate that there is no single breath marker for hyperoxia or oxidative stress. Instead, our analysis indicates that a collection of certain molecules or molecule classes such as straight-chain alkanes or methyl alkanes are exhaled in larger quantities, regardless of the type of hyperoxic exposure.

## 3. Discussion

After collection and analysis of GC-MS data from five studies on VOCs in exhaled breath after hyperbaric hyperoxic exposure, 41 compounds were identified, of which 12 molecules were excluded as contaminants after further analysis. The 29 remaining compounds consisted generally of straight-chain alkanes and methyl alkanes, and are associated with cell membrane damage, pulmonary pathology, or inflammation. The vast majority of identified compounds (n = 25) were found in several separate studies, indicating that exhalation of these compounds is a common principle after hyperbaric hyperoxia and strengthens their collective causality.

When comparing the results of this study with the excluded paper by Van Ooij et al. [11] on POT after a 100% oxygen dive, only two VOCs (out of a total of five molecules in the Van Ooij et al. study) are found in both studies. It is unclear to us why Van Ooij identified just five compounds compared with the larger finds of the included studies, but this can be the result of substantial improvements in GC-MS techniques since the publication of the Van Ooij et al. study. Nevertheless, both the Van Ooij et al. study and our included studies show that the majority of observed VOCs are methyl alkanes. This similarity helps to confirm our findings and strengthens the belief that there is no single marker for POT, but rather a collection of certain molecules or molecule classes that are exhaled in larger quantities. As mentioned in the Materials and Methods section, the Van Ooij et al. study was not included in our library because their GC-MS analysis was performed by a different laboratory.

To our knowledge, this is the first systematic reanalysis of studies that combined exhaled breath markers after hyperbaric and hyperoxic exposures in healthy individuals. All included studies had a comparable methodology for acquiring the breath samples, the GC-MS analysis was performed by the same laboratory and machine, and similar scripts in R were used by the same researcher to process the data. Therefore, a high degree of resemblance between the studies could be achieved, and data from various publications could be pooled while limiting confounders.

Several limitations of the present study need to be addressed. First, the included studies focused on finding signs of POT but all subjects were well-within acceptable limits for hyperoxic exposure, and except for some subjects in the COMEX-30 study none had subjective signs of POT. Interestingly, Willoughby et al. [16] largely identified the same compounds as this study when they exposed their test subjects to far greater hyperbaric hyperoxic exposures, during which 58% of the subjects experienced clinical signs of POT. This suggests that the same compounds and molecule classes can be found in symptomatic POT.

Just five studies were selected to compose the VAPOR library. It was decided to only include research from the same GC-MS laboratory with similar sampling and data analysis methods, which greatly facilitated the combining of the results and can provide internal validation of the results. Thus, we only included the most recent studies carried out by the research group of the Royal Netherlands Navy Diving and Submarine Medical Centre and excluded the work of Van Ooij et al. [11] and Willoughby et al. [16]. To our knowledge, other studies with comparable designs and endpoints have not been published. However, it may be that other POT-related studies are kept under military embargo or other restrictions and therefore have not (yet) been published. Nonetheless, we encourage other research groups to validate and expand this library with their data.

The test subjects of the included studies were part of a homogenous group of military divers and medical personnel. The majority were men between 25 and 45 years old, and all had to pass strenuous medical tests as part of the mandatory yearly medical examination of occupational diving and hyperbaric-related personnel, ensuring excellent baseline health and fitness. Furthermore, the test subjects had no prior hyperbaric exposure or other pulmonary irritants such as tobacco use and were not recently affected by a respiratory tract infection. This makes the results of this study highly relevant and suitable for the military and occupational diving population, but the results may not apply equally to other groups, such as patients receiving hyperbaric oxygen therapy or undergoing prolonged ventilation with an increased pO2. Thus, the VOCs after hyperbaric hyperoxic exposure in these populations should also be evaluated.

Also, not all significantly varying ion fragments could be identified with sufficient certainty. For example, at a retention time of 995 s, an ion cluster displayed a similarity of 92% for undecane (C11H24) and decane (C10H22), and 91% for dodecane (C12H26) in one of the studies. All these compounds are straight-chain alkanes, have been associated with pulmonary pathology, could be part of a cell membrane destroyed by lipid peroxidation, and have comparable similarity percentages. Thus, if the most viable compound could not be established with plausible certainty, it was decided to identify the ion cluster as a straight-chain alkane with its probable carbon length, collect these clusters in a separate table (Table 3), and mark the individual contesting compounds in Table 2.

As mentioned in the Materials and Methods section, to keep the library compact and comprehensible, only the most crucial characteristics of the identified compounds are included in Table 2, such as the CAS registry number, the molecular mass, and similar molecules with comparable ion profiles and high similarity for the associated retention times. This last category was added because when identifying some ion clusters, a high number of molecules in the NIST library had comparable similarity percentages. Other characteristics, although no less important, such as molecular formula, associated diseases, or their 2-D structures, were not included to keep the table comprehensible and brief by only including the most important traits for identification after GC-MS analysis.

Some VOCs identified in our analysis were not mentioned in their respective papers. This can occur if the compound was originally considered a contaminant but has been found in other studies in association with lung damage or pathology since the publication of the original study. For example, butyl acetate was discarded as a food additive in some initial studies by Wingelaar et al. since it was found after the test subjects were allowed to eat [8,12]. However, in de Jong et al. [10], this compound was identified before the subjects were allowed to eat; in other studies, an association was discovered in patients with asthma, cystic fibrosis, certain types of breast cancer, and a smoking habit [17,18,19]. Another possibility is that at a certain retention time, a different compound with comparable similarity was chosen by the author. This can be seen with 2,4-dimethylhexane and 3-methylheptane. The former was identified in the included studies as the compound at a retention time of 550 s [8,12,13]. Nevertheless, since the latter was equally similar to the targeted ion cluster, has been identified in more studies on reactive oxygen species and pulmonary damage, and is part of the macromolecular structure of the cell membrane, we decided in this analysis that this was the most viable compound [20,21,22].

As mentioned in the Results section, the majority of identified VOCs are straight-chain alkanes (n = 11), followed by methyl alkanes (n = 6). Most hydrocarbons, such as unbranched alkanes and alkenes, likely originate from the destruction of cell membranes by reactive oxygen species due to hyperoxia [23,24,25]. This process, called lipid peroxidation, damages the lipid bilayer of the cell membrane, leading to cell destruction or apoptosis. The polyunsaturated fatty acids in cell membranes are particularly susceptible to peroxidation because of the various relatively unstable methylene bridges and can break at various points depending on the location of the methylene bridge; these breakages result in alkanes and alkenes of various lengths [26,27].

Methyl alkanes, aldehydes, cycloalkanes, and esters originate from other processes of oxidative stress, inflammatory responses, or metabolic products of cell destruction [25,28].

One can argue that the VOCs identified in this study are increasingly exhaled as a consequence of increased ambient pressure and not just hyperoxia. However, to cancel the effect of increased ambient pressure, one of the included studies by Wingelaar et al. [8] had a crossover design with a control group breathing compressed air (78% nitrogen, 21% oxygen, and 1% other gases) instead of 100% oxygen. This comparison demonstrated a distinctive rise in VOCs after the 100% oxygen dives compared with the control group breathing compressed air. In a normobaric experiment with an increased pO2, Phillips et al. [28] observed a significant rise in the level of alkanes and methyl alkanes after hyperoxic exposure. Therefore, we can assume that the VOCs found in the included studies are induced by hyperoxia and not just by the hyperbaric environment.

Although all the included studies used slightly different breathing gases during the hyperbaric exposure (100% oxygen or a 50:50 helium-oxygen mix with or without intermittent air breaks), all the breathing gases were considerably hyperoxic. Depending on the depth of the dive or hyperbaric chamber regimen, the resultant partial pressure for oxygen was 132 kPa or greater, which is far higher than the normal partial pressure of oxygen in ambient air of 21 kPa. Unfortunately, due to the relatively limited amount of collected data and multiple variables between the hyperbaric exposures, we could not establish proper dose-response dependencies. Additional studies are needed to determine these relationships between the different depths and duration of the dives, the hyperoxic breathing gases used, and their associated VOCs.

## 4. Materials and Methods

To compose the Volatilome-Associated Pulmonary Oxygen Response (VAPOR) library, data from multiple studies conducted by the research group of the Royal Netherlands Navy Diving and Submarine Medical Centre on VOCs after hyperbaric hyperoxic exposure were collected [8,9,10,11,12,13]. The incorporated data consisted of the GC-MS output after initial processing for peak detection, denoising and retention time alignment, as previously described in the original studies and based on the work by Smith et al. [29]. See Table 4 for details and characteristics of the included studies.

All interventional studies were approved by the Medical Ethical Committee of the University of Amsterdam and the Surgeon General of the Ministry of Defence. The observational studies without interventional aspects were part of regular day-to-day diving and hyperbaric activities; therefore, no prior permission was necessary according to national legislation. All procedures were carried out in accordance with the ICH GCP E6(R2) Good Clinical Practice guideline and with the Surgeon General’s authorization. All subjects provided written informed consent and were free to withdraw from the study at any time. No data obtained during the included studies were documented in the participants’ medical files, in compliance with national privacy legislation and European Data Protection Regulations (GDPR).

**Figure 1:**
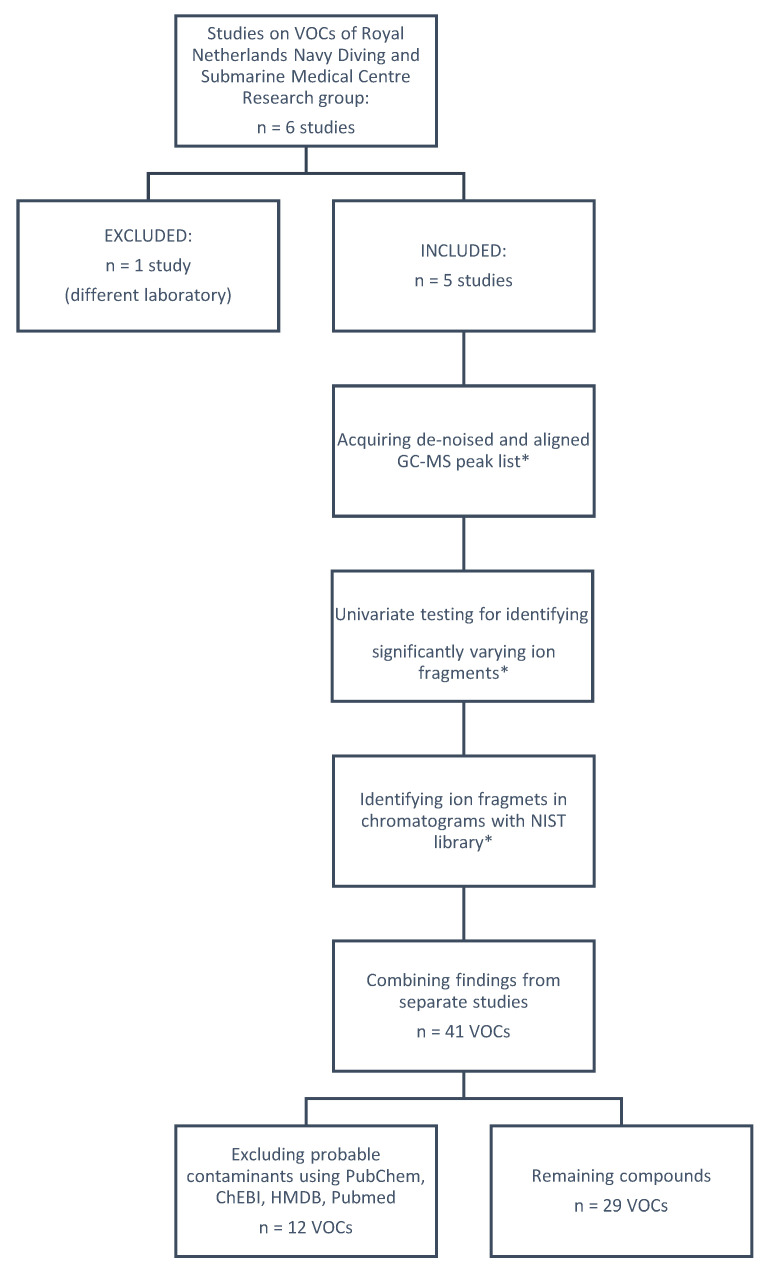
An overview of data acquisition, statistical analysis, and compound selection. * Procedures marked with an asterisk were performed for each study separately.

To minimize variations in data processing, reduce outcome bias, and warrant uniformity, all collected GC-MS data were statistically processed, analyzed, and identified without considering the final results of the respective studies. One study by Van Ooij et al. [11] was excluded since the breath samples were processed by a different laboratory and GC-MS. Four studies used an identical protocol for collecting samples and were carried out in a controlled laboratory setting. One study was performed during outdoor/field circumstances with a slightly different protocol, but the overall research protocol and sampling methods were similar to the studies in the laboratory setting. The field study had less longitudinal sample collection points due to operational limitations. See Figure 1 for an overview of the design, data collection, and analysis of this study.

### 4.1. Test Subjects and Preparation

All included test subjects were healthy, nonsmoking divers or trained hyperbaric medical personnel of the Royal Netherlands Navy. All subjects had to be medically cleared for diving and hyperbaric exposure in accordance with the Netherlands Ministry of Defence diving medical fitness requirements, which are based upon Dutch national policy on medical standards for occupational diving [30]. Exclusion criteria consisted of a recent respiratory tract infection, including COVID-19, or consumption of two or more alcoholic beverages per day. Furthermore, no hyperbaric exposure was allowed up to 72 h before the trials, and no alcohol could be consumed on the day before the trials. In addition, no strenuous physical exercise was allowed on the day before the laboratory trials. This criterion was different in a subgroup of the field trial, where seven divers from the Marine Corps had just completed a physically strenuous 5-day operational exercise.

Another difference between the field and laboratory studies was the subjects’ diet during the day of the trials. In the field setting, no food was consumed until after the last measurement. In the laboratory setting, eating was stimulated between test moments to prevent alterations in metabolism due to fasting. To minimize the effects of exogenous contamination of the breath samples due to different kinds of food, all test subjects had a uniform diet of bread, marmalade, and water. However, no eating was allowed within 1 h before measurements to further minimalize contamination of the breath samples.

### 4.2. Hyperbaric Hyperoxic Exposure

Two studies were performed after in-water dives, one in the wet compartment of the Medusa diving simulator (Haux Life Support, Karlsbad, Germany) and the other in a freshwater lake [8,13]. The other three studies were dry hyperbaric exposures in either the recompression chamber (Haux Life Support, Karlsbad, Germany) of the Royal Netherlands Navy Diving and Submarine Medical Centre or the Boerema chamber (Werkspoor/Boerema, Zuilen, the Netherlands) at Amsterdam University Medical Center, location AMC [9,10,12]. The test subjects in all the included studies breathed a hyperoxic gas mixture; four studies used 100% oxygen with or without short intermittent periods of breathing regular air, and in one study, the test subjects also partially breathed a hyperoxic mixture of helium and oxygen in a 50:50 ratio. The total exposure times ranged from 1 h to 7 h 30 min, with the depth (or the equivalent pressure in the studies that used a recompression chamber) varying between 3 and 30 m of seawater. The in-water dives were at 9 m for 1 h and 3 m for 3–4 h, and in these studies, the subjects were constantly breathing 100% oxygen. The exposures in the dry hyperbaric chamber were identical to frequently used treatment tables for divers with decompression illness or patients needing hyperbaric oxygen therapy: a modified version of the US Navy Treatment Table 6, a COMEX-30 treatment table, and a typical hyperbaric oxygen treatment table. See Table 4 for further details of the dive profiles of all the included studies.

### 4.3. Sample Collection and Analysis

Breath sampling protocols were also similar in the included studies; the test subject’s breath samples were collected for GC-MS analysis just before and at 30 min, 2 h, and 4 h after the hyperbaric exposure. One laboratory study collected additional samples at 1 and 3 h postexposure, while the field trial collected only the 30 min and 2 h postexposure samples. These additional samples were included in the VAPOR library.

To minimize environmental contamination, the sampling procedures started with the subject breathing through an inspiratory VOC filter (Honeywell, Charlotte, NC, USA) for 5 min. Thereafter, a nonelastic sampling balloon (Globos Nordic, Naestved, Denmark) was attached to the expiration aperture of the filter setup, and the subject fully exhaled into the balloon. The balloons were then mechanically sealed. Via a separate opening, 500 mL of exhaled air was pumped by an automatic gas pump (Gastec, Kanagawa, Japan) through a steel collection tube (Tenax GR 60/80, Camsco, Houston, TX, USA) for 2 min. All materials, including the tubing and one-way valves used in the different tests were identical in all the included studies. In all studies, environmental air was sampled to identify possible contaminants, such as from the oxygen masks or dive set tubing, food sources, and ambient air from the chamber.

The GC-MS machine (GC-MS QP2010, Shimadzu, Japan) of the Amsterdam University Medical Center was used to analyze the collected samples following an identical protocol in all the studies. In brief, the collection tubes were heated using a thermal desorption unit (Markes, Sacramento, CA, USA) to 250 °C for 15 min at a flow rate of 30 mL/min. The evaporated VOCs were captured in a cold trap at 10 °C and reheated rapidly to 300 °C for 1 min, and then transported via splitless injection into a 30 m gas chromatography column of 0.25 mm (Restec, Bellefonte, PA, USA) at 12 mL/min. An electron ionization of 70 eV was used to ionize the molecules, which were then identified by a quadrupole spectrometer with a scan range of 37–300 Da.

### 4.4. Statistical Methods, Data Analysis, and Identification

To identify the ion fragments in the breath samples that were significantly different (*p* < 0.05) between baseline and postexposure measurements per retention time, a Wilcoxon signed-rank test was used in R Statistical Software (v4.0.3; R Core Team 2021) combined with R-packages MBESS (v4.9.0; Kelley 2022), sva (v3.20.0; Leek and Storey 2008), and rstatix (v0.7.0; Kassambara 2021). After this selection through univariate testing, the ion fragments were sorted on their retention time. All retention times that yielded three or more statistically significant ion fragments were reviewed using the chromatograms, after which a similarity search and tentative identification based on the NIST webbook library (National Institute of Standards and Technology, National Bureau of Standards; US Department of Commerce, Gaithersburg, MD, USA) were carried out using GC-MS identification software (GC-MS Solution version 4.52, Shimadzu Corporation, Kyoto City, Japan). For this step, the five chromatograms with the highest peak intensity for the selected retention time were selected. Per chromatogram, the top five compounds with the highest similarity scores were selected for further processing. In consultation with the other authors of this study, and similar to what is used in other studies on VOC identification, 80% similarity was chosen as a cutoff point [20,31]. From this selection, the average percentage of each compound over multiple chromatograms was established, and the three highest overall scoring compounds were nominated in Table 2. When the difference between the third and subsequent compounds was ≤2%, these runner-up compounds were also included in Table 2.

The compound with the highest overall similarity score was noted in the first column of the table under the “Name” header. If the similarity comparison produced multiple eligible compounds, such as isomers or other similar molecules, additional chemical and biological characteristics and previous citations were collected and examined to choose the most likely molecule. The discarded molecules were noted in the “Matching Ion Profile” column. If further analysis could not establish the most viable molecule, the unidentified VOC and its probable chemical composition was noted in Table 3, and the molecules were recorded individually in the VAPOR library in Table 2.

Additional input for identifying VOCs such as associated diseases or CAS registration numbers [32] for the construction of Table 1 and Table 2 was collected using PubChem [33], the NIST library [34], the Human Metabolome Database (HMDB) [35], the Chemical Entities of Biological Interest database (ChEBI) [36], and Pubmed [37].

The compounds were identified as contaminants if they were also found abundantly in the environmental samples, or none of the final selected molecules had any association with the human volatilome or were established as being nonhuman in PubMed, HMDB, or ChEBI.

### 4.5. Library Design

To keep the combined VAPOR library comprehensible, only characteristics that are frequently used to identify compounds are included. This information comprises the name, CAS registration number, molecular weight, isomers or other similar molecules, and references to the corresponding papers.

## 5. Conclusions

The VAPOR library contains 29 compounds associated with hyperbaric hyperoxic exposure and greatly reduces the resources required for future analysis and identification of breath samples associated with the pulmonary response to these environments. The VOCs that could not be identified with sufficient certainty are summarized separately, with details of their most likely identities.

Within this library, a substantial number of molecules were identified in the majority of the included studies. Furthermore, the nonincluded studies on VOCs with similar design, protocols, and endpoints or with subject reporting clinical signs of POT, identified largely the same compounds and molecule classes. Together, these findings indicate that the compounds in the VAPOR library are strongly associated with pulmonary reactions to hyperbaric hyperoxia or even POT. However, no VOC was found in all the individual studies, indicating that no single breath marker for hyperbaric oxidative stress exists; rather, it is likely that a collection of molecules or molecule classes such as alkanes or methyl alkanes are exhaled in larger quantities during hyperbaric hyperoxia, regardless of the type of hyperoxic exposure.

We encourage other research groups to validate our findings and possibly expand this library with additional data.

## Figures and Tables

**Table 1:** Excluded compounds.

Name	CAS No.	Molecular Weight	Matching Ion Profiles	References
1,2-Dichloropropane	78-87-5	112	1,1-Dichloropropane	[8,9,12]
Toluene	108-88-3	92	1,3,5-Cycloheptatriene; 2,5-Norbornadiene	[9,13]
4,5-Dimethyl-1,3-dioxane	1779-22-2	116	2,6-Dimethyl-1,4-dioxane; 2,5-Dimethyl-1,4-dioxane	[10]
Hexamethylcyclotrisiloxane	541-05-9	222		[8]
Ethylcyclohexane	1678-91-7	112		[12]
Ethylbenzene	100-41-4	106	O-Xylene; P-Xylene	[8,13]
Hexanenitrile	628-73-9	97	Heptanonitrile	[10]
Cyclohexanone	108-94-1	98	2-Methylcyclopentanone	[8]
Isopropylbenzene	98-82-8	120		[8]
2-Ethyl-1-octene	51655-64-2	140	3-Methyl-2-nonene; 3-Methyl-6-methyleneoctane	[8]
(Z)-beta-Ocimene	3338-55-4	136	Trans-Ocimene; Alpha-Ocimene; 3-Isopropenyl-5,5-dimethylcyclopentene	[8]
1-Pentadecene	13360-61-7	210		[12]

**Table 2:** The Volatilome-Associated Pulmonary Oxygen Response (VAPOR) library.

Name	CAS No.	Molecular Weight	Matching Ion Profiles	References
Isoprene	78-79-5	68	1,3-Pentadiene; Ethylidenecyclopropane	[8,10]
Hexane	110-54-3	86		[12,13]
2,4-Dimethylpentane	108-08-7	100	iso-Butoxyamine *; Isopentane *	[9,13]
Ethyl acetate	141-78-6	88	4-Hydroxy-2-butanone *; Ethyl pyruvate *; Methylazoxymethanol acetate *	[9,12]
Cyclohexane	110-82-7	84	Methylcyclopentane; 2-Methyl-1-entene	[8,12,13]
Propyl acetate	109-60-4	102	Isopropyl acetate; Dipropyl sulfite *	[10]
Methylcyclohexane	108-87-2	98	2,3-Dimethyl-2-pentene *; (E)-3,4-Dimethyl-2-pentene *; (Z)-3,4-Dimethyl-2-pentene *; trans/cis-1-Ethyl-3-Methylcyclopentane *	[8,9,10,12]
3-Methylheptane	589-81-1	114	2,4-Dimethylhexane; 3-Ethyl-2-methylhexane	[8,10,12,13]
3-Methyleneheptane	1632-16-2	112	3-Methyl-1-heptene *; 2-Ethylhexyl acrylate *; 2-Octene *	[9,10,12]
Octane	111-65-9	114	2,4-dimethylheptane; Nonane	[8,10,12]
Butyl acetate	123-86-4	116	Isobutyl acetate; Hexyl acetate *	[8,9,10,12]
Nonane	111-84-2	128	3,4-dimethylheptane; 2-Methylnonane *; Heptane; Decane	[8,9,10,12]
3-Methylnonane	5911-04-6	142	2,6-dimethyloctane *; 4-Methyl-1-decene *; 3-Ethyl-5-methylheptane *	[8,10,12,13]
1-Decene	872-05-9	140		[8,12]
Decane	124-18-5	142	2-Methylnonane *; Nonane; 4-Ethyloctane *; Undecane	[8,9,10,12]
2-Butyl-1-octanol	3913-02-8	186	2-Methyloctan-1-ol *; 4-Methyl-2-propyl-1-pentanol *; 3,4-Dimethyl-1-decene *; 2,3,5,8-Tetramethyldecane *	[8]
3-[(1,1-Dimethylethoxy)methyl]heptane	83704-03-4	186	2,2-Dimethyl-4-decene *; (Z)-, 4-Octanol, propanoate *	[8]
2-Methylundecane	7045-71-8	170	4,6,8-Trimethyl-1-nonene *; 2,3,5,8-Tetramethyldecane *	[8,10,12]
Undecane	1120-21-4	156	Decane; Dodecane	[8]
3,7-Dimethyldecane	17312-54-8	170	5-butylnonane; Hexadecane	[8]
Nonanal	124-19-6	142	Decanal; Dodecanal; Undecanal *; (E)-2-Nonen-1-ol *	[8,9,10,12]
Dodecane	112-40-3	170	2-methylundecane; Decane; Tridecane; Undecane; Hexadecane	[8,10,12]
Tridecane	629-50-5	184	2,3,5,8-Tetramethyldecane; 1-Iodo-2-methylundecane; Dodecane; Pentadecane	[8,9,10,13]
Decanal	112-31-2	156	1-Nonadecanol *; 1-Eicosanol *; Dodecanal; (E)-2-Decen-1-ol *	[8,9]
Tetradecane	629-59-4	198	3-Methylundecane; Tridecane; Hexadecane	[8,10,12]
3-Methylundecane	1002-43-3	170	2,6,10-Trimethylpentadecane *; 3,5-Dimethyldodecane *; 3-Methyltridecane *	[8,12]
Pentadecane	629-62-9	212	Tridecane; Nonadecane; Dodecane; Tetradecane; Eicosane; Hexadecane	[8,12]
Hexadecane	544-76-3	226	Dodecane; Pentadecane; Tetradecane	[8,12,13]
Nonadecane	629-92-5	268	5-(2-Methylpropyl)nonane *; 2,6,11-Trimethyldodecane *	[8,13]

* Identified as a contaminant in the literature.

**Table 3:** Unidentified VOCs due to multiple comparable similarity scores; all these compounds are straight-chain alkanes.

Characteristics	Matching Ion Profiles	Reference
Straight-chain alkane; 10–12 carbon molecules	Undecane; Decane; Dodecane	[8]
Straight-chain alkane; 11–16 carbon molecules	Dodecane; Tridecane; Hexadecane; Undecane	[8]
Straight-chain alkane; 12–16 carbon molecules	Dodecane; Tridecane; Hexadecane	[8]
Straight-chain alkane; 12–15 carbon molecules	Pentadecane; Tetradecane; Dodecane	[8]
Straight-chain alkane; 15–20 carbon molecules	Eicosane; Hexadecane; Pentadecane	[8]

**Table 4:** Studies and their characteristics included in the VAPOR library.

Included Studies	No. of Subjects (No. of Samples)	Hyperbaric Exposure *	Breathing Gas ^†^
Wingelaar TT et al. [8]	12 (72)	60 min in-water	100% O_2_ (60 min)
		193 kPa	
Wingelaar TT et al. [12]	10 (171)	10 × 95 min dry	100% O_2_ (80 min)
		253 kPa	Air ^‡^ (15 min)
Wingelaar TT et al. [13]	4 (12)	240 min in-water	100% O_2_
		132 kPa	
	7 (14)	180 min in-water	100% O_2_
		132 kPa	
de Jong FJM et al. [10]	14 (56)	285 min dry	100% O_2_ (240 min)
		283 kPa (105 min) 192 kPa (180 min)	Air ^‡^ (45 min)
de Jong FJM et al. [9]	10 (40)	450 min dry	Heliox 50/50 ^§^ (135 min)
		405 kPa (90 min) 345 kPa (60 min) 283 kPa (90 min) 192 kPa (210 min)	100% O_2_ (255 min) Air ^‡^ (60 min)

* The duration of exposure, type of exposure (dry/chamber vs in-water/submerged), pressures used (if multiple pressures were used, the corresponding times including descend times are mentioned). ^†^ If multiple breathing gases were used, the corresponding total breathing times are mentioned. ^‡^ Air = 21% N_2_, 79% O_2_. ^§^ Heliox = 50% helium and 50% O_2_.

## Data Availability

The data presented in this study are available on reasonable request from the corresponding author.
